# Supplementary material for: Prostaglandin F2α Affects the Cycle of Clock Gene Expression and Mouse Behavior
Source: Int J Mol Sci. 2024 Feb 2;25(3):1841. doi: 10.3390/ijms25031841 (PMC10855224; doi:10.3390/ijms25031841)
Supplement: Supplementary file 1 [file ijms-25-01841-s001.zip › ijms-2808901-supplementary.pdf]

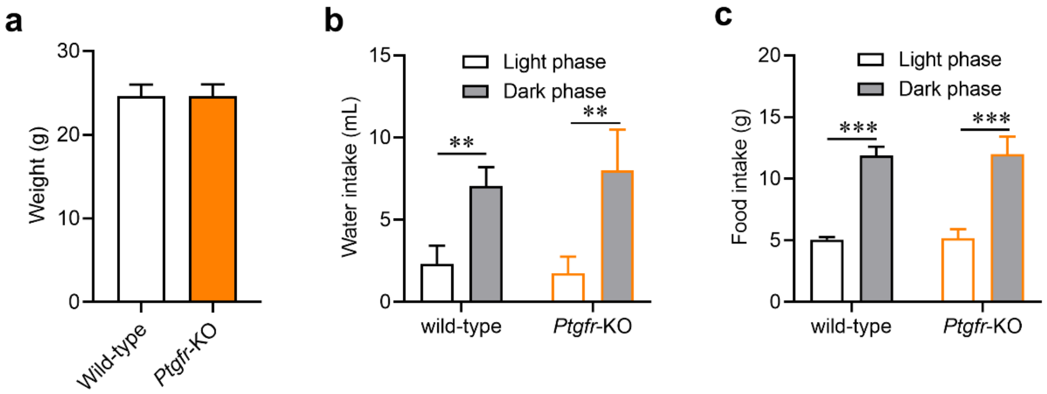

Figure S1. Body weight, water, and food intake in 8-week-old wild-type and *Ptgfr-KO* mice. (a) Body weight in 8-week-old wild-type and *Ptgfr-KO* mice (n = 6). There was no significant difference between wild-type and *Ptgfr-KO* mice (t-test, $p = 0.975$). (b) Diurnal rhythm of food intake in wild-type and *Ptgfr-KO* mice (n = 4). Food intake (in grams) was measured during light phase and dark phase. **$p < 0.01$; significant difference between two groups (two-way ANOVA with Tukey's post-hoc test; Genotype: $F_{1,12} = 0.0575$, $p = 0.815$; Time: $F_{1,12} = 50.23$, $p < 0.001$; Interaction: $F_{1,12} = 0.930$, $p = 0.354$). (c) Diurnal rhythm of water intake in wild-type and *Ptgfr-KO* mice (n = 4). Water intake (in mL) was measured during light phase and dark phase. ***$p < 0.01$ significant difference between two groups (two-way ANOVA with Tukey's post-hoc test; Genotype: $F_{1,12} = 0.0727$, $p = 0.792$; Time: $F_{1,12} = 245.3$, $p < 0.001$; Interaction: $F_{1,12} = 0.00053$, $p = 0.982$).

Table S1. The results of the cosinor analysis.

	Group	Amplitude	Mesor	Acrophase (h)	Period (h)	P value
Figure 2a	Wild-type	0.454	0.609	3.89	18.8	***<0.001
	Ptgfr-KO	0.297	0.607	3.41	18.1	***<0.001
Figure 2b	Wild-type	0.447	0.504	7.27	24.6	***<0.001
	Ptgfr-KO	0.374	0.468	8.00	22.1	***<0.001
Figure 2c	Wild-type	0.613	0.345	8.99	23.7	***<0.001
	Ptgfr-KO	0.529	0.237	13.60	25.6	***<0.001
Figure 2d	Wild-type	0.311	0.643	13.00	25.4	**<0.01
	Ptgfr-KO	0.238	0.542	13.80	25.9	**<0.01
Figure 3a	Wild-type	0.404	0.552	13.13	24.6	***<0.001
	Ptgfr-KO	0.636	0.560	13.64	23.6	***<0.001
Figure 3b	Wild-type	0.456	0.384	13.33	24.2	***<0.001
	Ptgfr-KO	0.351	0.271	10.33	24.2	***<0.001
Figure 3c	Wild-type	0.526	0.381	22.00	21.4	0.12
	Ptgfr-KO	0.502	0.438	22.27	20.3	***<0.001
Figure 3d	Wild-type	0.424	0.452	16.00	25.1	***<0.001
	Ptgfr-KO	0.732	0.617	14.26	18.0	***<0.001
Figure 3e	Wild-type	0.193	0.660	11.40	24.6	*<0.05
	Ptgfr-KO	0.424	0.684	4.36	18.9	***<0.001
Figure 3f	Wild-type	0.483	0.320	6.81	21.4	**<0.01
	Ptgfr-KO	0.483	0.318	5.06	20.1	***<0.001
Figure 4a		0.273	0.631	20.90	25.1	***<0.001
Figure 4b		39.8	183.1	14.00	16.0	0.0607
Figure 4c		6.83	14.7	20.87	10.6	***<0.001
Figure 5a		31.9	49.3	13.34	18.4	*<0.05
Figure 5b		36.3	64.1	17.88	24.0	***<0.001

Table S2. Primer sets for PCR analysis of gene expression

Gene	Accession number	F or R	Primers
Mouse *Per1*	NM_011065	Forward<br>Reverse	5′- CCAGATTGGTGGAGGTTACTGAGT -3′<br>5′- GCGAGAGTCTTCTTGGAGCAGTAG -3′
Mouse *Per2*	NM_011066	Forward<br>Reverse	5′- GACTGCGACGACAATGGGAA -3′<br>5′- TTTGGCAGACTGCTCACTACT -3′
Mouse *Bmal1*	NM_007489	Forward<br>Reverse	5′- ACGACATAGGACACCTCGCAGA -3′<br>5′- CGGGTTCATGAAACTGAACCATC -3′
Mouse *Cry1*	NM_007771	Forward<br>Reverse	5′- AGGGAACCCCATCTGTGTTC -3′<br>5′- TGGTGCATTCCAAGGATCGT -3′
Mouse *Cry2*	NM_009963.4	Forward<br>Reverse	5′- GGCACCTGTGATGTCCATGTTTA -3′<br>5′- GCAGAATTAGCCTTTGCTCCTGA -3′
Mouse *Dbp*	NM_016974	Forward<br>Reverse	5′- CCGTGGAGGTGCTAATGACCT -3′<br>5′- CCTCTGAGAAGCGGTGTCT -3′
Mouse *Ptgfr*	NM_008966.3	Forward<br>Reverse	5′- GGAAAGAGAGGTGGAACCCG -3′<br>5′- CAACTGTGCAGTCTCGGAGT -3′
Mouse *β-Actin*	NM_007393	Forward<br>Reverse	5′- GACGGCCAGGTCATCACTATT -3′<br>5′- TACCACCAGACAGCACTGTGTT -3′
